# Evaluating Fidelity in Home-Visiting Programs a Qualitative Analysis of 1058 Home Visit Case Notes from 105 Families

**DOI:** 10.1371/journal.pone.0036915

**Published:** 2012-05-18

**Authors:** Thomas Saïas, Emilie Lerner, Tim Greacen, Elodie Simon-Vernier, Alessandra Emer, Eléonore Pintaux, Antoine Guédeney, Romain Dugravier, Susana Tereno, Bruno Falissard, Florence Tubach, the CAPEDP Study Group, Anne Revah-Levy

**Affiliations:** 1 Laboratoire de Recherche, Etablissement Public de Santé Maison Blanche, Paris, France; 2 Service de Pédopsychiatrie, Hôpital Bichat Claude-Bernard, Paris, France; 3 Institut National de la Santé et de la Recherche Médicale, U669 PSIGIAM, Paris, France; 4 Unité de Recherche Clinique Paris-Nord, Hôpital Bichat Claude-Bernard, Paris, France; 5 Institut National de Prévention et d’Education pour la Santé, Saint-Denis, France; 6 Département de Psychologie, Université du Québec à Montréal, Montréal, Canada; 7 Labosdif, Universitè de Trento, Rovereto, Italy; 8 Centre de Soins Psychothérapeutiques de Transition pour Adolescents, Hôpital d’Argenteuil, Argenteuil, France; 9 Université Paris-Sud, Université Paris-Descartes, Paris, France; The University of Adelaide, Australia

## Abstract

**Objective:**

Implementation fidelity is a key issue in home-visiting programs as it determines a program’s effectiveness in accomplishing its original goals. This paper seeks to evaluate fidelity in a 27-month program addressing maternal and child health which took place in France between 2006 and 2011.

**Method:**

To evaluate implementation fidelity, home visit case notes were analyzed using thematic qualitative and computer-assisted linguistic analyses.

**Results:**

During the prenatal period, home visitors focused on the social components of the program. Visitors discussed the physical changes in pregnancy, and psychological and social environment issues. Discussing immigration, unstable employment and financial related issues, family relationships and dynamics and maternity services, while not expected, were found in case notes. Conversely, health during pregnancy, early child development and postpartum mood changes were not identified as topics within the prenatal case notes. During the postnatal period, most components of the intervention were addressed: home visitors observed the mother’s adaptation to the baby; routine themes such as psychological needs and medical-social networks were evaluated; information on the importance of social support and on adapting the home environment was given; home visitors counseled on parental authority, and addressed mothers’ self-esteem issues; finally, they helped to find child care, when necessary. Some themes were not addressed or partially addressed: health education, child development, home environment, mother’s education plans and personal routine, partner support and play with the child. Other themes were not expected, but found in the case notes: social issues, mother-family relationship, relation with services, couple issues, quality of maternal behavior and child’s language development.

**Conclusions:**

In this program, home visitors experienced difficulties addressing some of the objectives because they gave precedence to the families“ urgent needs. This research stresses the importance of training home visitors to adapt the intervention to the social, psychological and health needs of families.

## Introduction

Home-visiting programs have become one of the most popular early childhood interventions. These programs serve more than 500,000 families in the United States [1] and are of growing interest in Europe as an additional benefit to welfare state prevention systems [2]. However, in recent reviews of home-visiting programs, only half of the reviewed programs had a significant and positive impact on the participating children [3], [4]. Whether these mixed findings can be attributed to differences between intervention methods or population recruitment criteria, or to insufficient fidelity to evidence-based program intervention protocols, is not clear.

One of the main purposes of home-visiting programs is to reduce the impact of social stress on the mental health of vulnerable families [5]–[8]. Early childhood intervention services vary depending upon the target population. Typically early childhood interventions follow a home-visiting program model, which provides emotional support, psychoeducation and case management to the families on a weekly or monthly basis, with services beginning as early as the prenatal period and ending as late as the child’s fifth birthday [9]. Most of these programs draw upon attachment [10] theory, self-efficacy [11] theory and human ecological systems [12] theory as a basis for their interventions [13].

Although home-visiting programs share common features, visiting clients’ homes remains a method for delivering a service rather than a service in itself [14]. Understanding the overall impact of home-visiting programs is a challenge for researchers because a variety of program models have been implemented in the past [15]. Most program evaluations have used quantitative measures targeting child outcomes.

Program implementation, i.e. applying a program protocol in practice, is currently an important yet very recent [16] focus of intervention research as it determines a program’s effectiveness in accomplishing its original goals [17]. In a recent review on psychosocial interventions, Perepletchikova, Treat & Katzdin [18] have revealed that only 3.5% of these researches documented accurately fidelity. Assessing program implementation addresses issues such as recruitment rates [19], attrition rates [20], program dosage [21] and discrepancies between the services which the program had initially intended to deliver and the services that were actually delivered. To evaluate *how well* a program was implemented, researchers used specific instruments such as questionnaires [18], self-reported measures (from participants and/or practitioners) [22], focus groups [23], in vivo observation [24], video recording [25], [26] or audio recording [27]. Though globally accurate, all strategies proved to be incomplete or biased. In a recent review, Breitenstein et al. pointed out the self-desirability bias in self-reported measures, the cost and the reactivity due to in vivo or video observations and the lack of environment assessment in audio recordings [28]. More methodological research is needed to develop sensitive yet accurate measures in preventive interventions. In home-visiting research, because of the centrality of the home visitor-family relationship, researchers used post home visit fidelity evaluation, with home visitors self-reports, or participants/providers focus groups.

In a qualitative study (tape recorded nurses’ case notes) of the challenges experienced by professionals working in the Nurse Family Partnership (NFP) program, Kitzman, Cole, Yoos, & Olds [23] emphasized the disparity between the program’s objectives and the home visitor’s efforts to address the needs of the families. They argue that program objectives often failed to be implemented because developing the nurse-family relationship (gaining and maintaining access to the family, identifying relevant actors, balancing nurse - client responsibilities) became an overwhelming priority. Delivering the intended services was one of the nine challenges experienced by home visitors and identified by the authors. In the Memphis NFP trial comparing the interventions of non-professional home visitors to paraprofessionals, Hiatt, Sampson & Baird [29] found through the analysis of implementation data (home visit case notes, supervision data) that the smaller effect of the paraprofessionals’ intervention was partially explained by the focus on situational and environmental issues instead of on the parenting curriculum, which had been perceived as “foreign and unnecessary”. Darius Tandon, Mercer, Saylor & Duggan [30] found though a series of focus groups with paraprofessional home visitors that home visitors working with vulnerable populations experienced conflict between responding to the families’ urgent needs and strictly adhering to program protocol.

In a longitudinal mixed methods study (families and home visitor case studies, focus groups, videotaping and interviews), Hebbeler & Gerlach-Downie [15] argued that the *Parents as Teachers* program failed to achieve its initial goals because the professionals prioritized social support over adhering to program protocol, which emphasized behavioral change. Hebbeler & Gerlach-Downie agree with Gurlanick’s statement that, when evaluating a home-visiting program, the key question is not “does it work?” but “what works for whom under what circumstances?” [15], [31]. Following these statements Woolfolk & Unger [32] conducted a qualitative study (interviews of mothers) of the *Parents as Teachers* program and found that home visits with low-income African American families varied from family to family despite the fact that the visits took place within the same program and following the same program model. Using open-ended interviews with the mothers in question, they found that families where mothers played an active role in shaping the relationship with the home visitor and the content of the visit, benefited more from the intervention. New studies to determine which program services best respond to the needs of particular populations will likely reveal further barriers to and facilitators of successful program implementation.

The following study seeks to evaluate the extent to which the manualised program guidelines were reflected in home visitor case notes in an early childhood intervention, the CAPEDP Project [Compétences Parentales et Attachement dans la Petite Enfance: Diminution des Risques Liés aux Troubles de Santé Mentale et Promotion de la Résilience] and to identify case note themes that did not figure in the program.

## Materials and Methods

### The CAPEDP Project

The CAPEDP Project took place in Paris, France, from 2006 to 2011. The project was developed to consolidate perinatal and early childhood mental health promotion services in Paris and its suburbs, by offering home visit support to families presenting demographic characteristics associated with a higher incidence of subsequent maternal postpartum depression and infant mental health problems: mothers had to present one or more of the following inclusion criteria to participate in the program: (1) having less than 12 years of schooling, (2) intending to raise the child without the father (3) being eligible for health care free of charge, due to lack of personal resources or income. The program aimed to reduce the incidence of maternal postpartum depression and infant mental health problems as well as to promote parenting skills, infant-mother attachment security and social and professional integration. A total of 440 pregnant, primiparous women under the age of 26 were recruited in maternity wards between 2006 and 2009. Median age of participants was 22 years; 28.3% of the sample were mothers intending to raise their child alone; 74.4% had less than 12 years education and 45.7% were eligible for free health care. 52.3% were born outside France [33]. CAPEDP has been registered to Clinical Trial with the number NCT00392847.

The visits were conducted by a team of nine psychologists. All were female and from 23 to 34 years of age when recruited. All home visitors received specific training in the CAPEDP service implementation protocol, which was backed up with a detailed training manual built around four periods in the baby’s life: prenatal period, 0 to 6 months, 6 to 15 months and 15 to 24 months. The program and its manual were largely based on the work of Weatherstone [34], the *Partners for a Healthy Baby* program [35], and the *Steps Towards Effective, Enjoyable Parenting* (STEEP™) program [36]. Visits began at the seventh month of pregnancy and continued until the child’s second birthday. Home visitors each visited from 15 to 35 families during the project. Of the 189 families who received one or more home visits, 125 had only one home visitor, while 54 families had two and 10 had three. Staff turn-over in the home-visiting team was the only reason for having more than one home visitor working with one family.

To evaluate to what extent the priorities of the home visitors as reflected in the home visit case notes reflect the intended processes of the intervention, we qualitatively analyzed the home visitor case notes. All home visit case notes were written by the home visitors themselves. It allowed us to evaluate both the frequency of themes that were declared as discussed during the home visit as well as the home visitor’s subjective perception of the visit.

The present qualitative study constitutes the first stage in evaluating the CAPEDP trial. Results from this trial will be published in the coming years, and discussed in regards to the services that were actually delivered to the participating families.

### Home Visit Case Notes

In the routine stage of the research (inclusion started in June 2007 and ended in January 2008), we randomly selected 10 to 12 families from the case load of each of the nine home visitors to participate in the current study. A total of 105 families were randomly selected. For the duration of two years (until June 2009), home visitors were asked to indicate, after each visit, the duration of the visit, the place where the visit was conducted (at home, in a public place, at hospital…) and the people present during the visit for each of the selected 105 families. Home visitors were then asked to write a brief report on the current family situation, the topics discussed during the visit and the relationship with the family. The 105 families received a total of 2,457 home visits from 2006 to 2010 and the home visitors collected a total of 1,058 case notes from 2007 to 2009.

**Figure 1:**
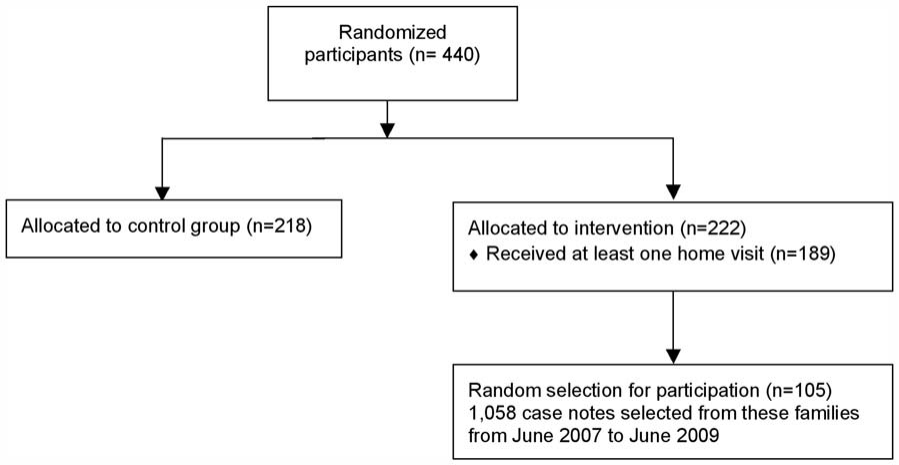
Stages leading to inclusion in the final sample.

Figure 1 synthesizes the stages leading to inclusion in the final analytic sample for the qualitative study.

### Participants

A total of 1,058 case notes were collected from 105 families, which represent 26.2% of the 4,034 home visits that took place during the 5-years program.

Each of these 105 families received an average of 23.4 home visits (1–63; SD = 14.5) from the 7^th^ month of pregnancy until the end of the program. This average number of home visits represents 53.2% of the number of home visits that the CAPEDP project had programmed per family. For these 105 families, on average 10.1 case notes (1–48, SD = 10.9) were written from the 7^th^ month of pregnancy until the end of the program.

Following the program intervention manual, case notes were divided into four chronological child age categories: prenatal, 0–6 months, 6–15 months and 15–24 months. Table 1 describes the total number of case notes, the mean length of a home visit, the percentage of home visits that took place outside the home and the percentage of visits where the father was present or with another person besides the parents present from the 1,058 home visit case notes.

**Table 1:** Characteristics of the 1,058 reported home visits.

	Prenatal	0–6 months	6–15 months	15–24 months	TOTAL
N case notes	289	369	262	138	**1058**
Mean Length of the visit (minutes)	70.9	69.6	71.2	65.1	**69.2**
% visits outside the residence	18.3	16.5	8.0	7.2	**12.5**
% visits with the father	12.8	15.2	11.1	13.0	**13.0**
% visits with another person	18.3	18.4	19.8	15.2	**18.0**

The demographic characteristics of the 105 families for whom case notes were collected were compared to those of all participating families. No significant differences between these two groups were found in terms of maternal age, percentage of single mothers, history of immigration, maternal and paternal education level, income, unplanned pregnancy, perceived health, and maternal attachment.

### Procedure and Data Analysis

Case notes were first classified into four child age categories and then randomly organized using Microsoft® Excel’s = RAND() function. The first 50 case notes from each of the four categories were extracted for thematic qualitative analysis. All 1,058 case notes from the 105 included families were used for the linguistic analysis.

#### Thematic qualitative analysis

The thematic analysis of the case notes was conducted using principles of grounded theory [37], [38]. The 4 readers performing the analysis of the case notes were mental health professionals, trained in qualitative analysis. They were not familiar with the program manual. Ten case notes from each of the four child age periods were coded by all analysts to create an initial template as a basis for subsequent analyses. Each reader then analyzed 50 case notes (200 case notes analyzed in total) to identify the primary themes. Content analysis involved inductive and deductive techniques. Topics emerging from case notes were compared to one another in order to identify broader themes from each of the four child age categories and across all categories. Discrepancies between coders were discussed. Thematic saturation, the point at which review of further data does not generate new categories [37], was attained between forty and fifty case notes, depending on the readers. Hence, all readers were asked to read 50 case notes (upper limit of saturation), in order to ensure total saturation for all periods. Extracts from the verbatim transcripts were translated in English and presented in the results section of this paper.

#### Computer-assisted linguistic analysis

A computer-assisted linguistic analysis using ALCESTE software [Analyse des Lexèmes Coocurrents dans un Ensemble de Segments de Textes, or Analysis of Co-occurring Lexemes in a Set of Text Segments [39]] was then performed for all case notes for each of the four child age periods. The ALCESTE computer-assisted analysis, recently used in several high-quality qualitative research publications (e.g. [40]), uses an algorithm to identify patterns found within a given text according to how often words appear together. It displays a categorization (classes of words frequently associated) which is then interpreted by the researcher, to provide meaning from these data.

Categories from the thematic analysis and the computer-assisted analysis were then compared to the CAPEDP manual for each intervention period. The purpose of these comparisons was to investigate to what extent the themes identified in case notes corresponded to instructions given by the project manual. For each child age period, we identified themes present in the case notes but not in the manual, and the themes absent from the case notes but targeted explicitly in the manual for that period. This information illustrates the extent to which the CAPEDP intervention was faithful to its initial model and guidelines.

## Results

Tables 2, 3, 4 and 5 present the categories identified from both thematic and computer-assisted analyses. To evaluate fidelity in program delivering, we have monitored the verbs associated with each theme. Verb-theme categories derived from the case notes were then compared to the manual’s guidelines, which had been organized into general themes for the purpose of this study.

**Table 2:** Prenatal period: categories from the intervention manual compared to themes identified in qualitative analysis of home visitor case notes.

Category		Program Manual Objectives	Thematicanalysis	ALCESTEanalysis	Additional (+) oromitted (−) thematic
Observe	Material needs	X	X	X	
Discuss	Physical and psychological changes during Pregnancy	X	X	X	
	Expectations of the baby	X	X	X	
	Presence of social support	X	X	–	
	Immigration, financial and employment issues	–	X	X	+
	Relationship with family	–	X	–	+
	Relations with maternity services	–	X	–	+
Inform	Delivery	X	X	X	
	Postpartum mood changes	X	–	–	−
	Support from partner	X	X	–	
	Importance of breastfeeding	X	X	–	
	Fetal development	X	X	X	
	First developmental stages	X	–	–	−
Counsel	Health during pregnancy	X	–	–	−
Do	Negotiate the objectives of the home visit	X	X	X	

**Table 3:** 0–6 months period: categories from the intervention manual and as developed at each stage of the qualitative analysis.

Category		Program manual objectives	Thematicanalysis	ALCESTEanalysis	Additional (+) oromitted (−) thematic
Observe	Psychological needs	X	X	X	
	Knowledge of child needs	X	X	–	
	Medical and Social network	X	X	–	
	Quality of parenting	–	X	–	+
Discuss	Couple needs	X	X	–	
	Job/Education-related needs	X	–	X	
Inform	Health education	X	–	–	−
	Feeding and sleep	X	X	X	
	Importance of partner support	X	X	–	
	Adaptation of home environment	X	–	–	−
	Baby’s early development	X	–	X	
Counsel	Parent-child interactions	X	X	X	
	Promote social support	X	X	–	
	Administrative needs	–	X	X	+
	Self-esteem	–	X	–	+
Do	Negotiate the objectives of the home visit	X	X	X	

**Table 4:** 6–15 months period: categories from the intervention manual and as developed at each stage of the qualitative analysis.

Category		Program manual objectives	Thematicanalysis	ALCESTEanalysis	Additional (+) or omitted (-) thematic
Observe	Psychological needs	X	X	–	
	Mother and Child Health Needs	X	X	X	
	Attachment quality	X	X	X	
	Medical and Social network	X	X	X	
Discuss	Couple needs	X	X	–	
	Educational plans	X	–	–	−
Inform	Importance of partner support	X	–	–	−
	Adaptation of home environment	X	–	X	
	Developmental stages	X	X	X	
Counsel	Promote self-esteem	X	X	–	
	Elaboration of personal goals	X	X	X	
	Parents-child interactions	X	X	X	
	How to set limits	X	X	X	
	Promote social support	X	X	–	
	Administrative problems	–	X	X	+
Do	Help to find child care	X	X	–	
	Negotiate the objectives of the home visit	X	X	X	

**Table 5:** 15–24 months period: categories from the intervention manual and as developed at each stage of the qualitative analysis.

Category		Program manual objectives	Thematic analysis	ALCESTE analysis	Additional (+) or omitted (−) thematic
Observe	Health needs	X	X	–	
	Mental health needs	X	X	X	
	Medical Social network	X	X	X	
	Language development concerns	–	X	–	+
Discuss	Representations of parental authority	X	X	X	
	Importance of social support	X	X	–	
	Feedback on intervention	–	X	X	+
	Social and professional situation	–	X	X	+
	2^nd^ pregnancy/2^nd^ child health	–	X	–	+
	Problems with romantic partner/Child’s Father	–	X	–	+
Inform	Importance of play	X	–	–	−
	Child development and autonomy	X	X	X	
Counsel	Organizing the schedule = the mother can take some time off for her/her couple	X	–	–	−
	Set limits for the child	X	X	X	
	Parent child interactions	X	X	X	
Do	Negotiate the objectives of the home visit	X	X	X	
	Network for social, health and mental health services	X	X	X	

### Prenatal Period

The main thematics that were to be addressed during this first period were (a) to negotiate the objectives of CAPEDP for each family, (b) to counsel the mother about health during pregnancy and to inform her about the main changes in post partum period and (c) to discuss the changes related to her pregnancy, her expectations regarding her future baby and the possibility to appeal to her social support network. Finally, the home visitors were asked to observe the material needs of the family and, if the situation was precarious, the visitor was asked to help the family to find financial or social resources before the delivery.

Table 2 synthesizes the results concerning home visits during the prenatal period. The results show that home visitors had a specific focus on the social components (e.g. observing material needs) of the program. Visitors also discussed the mother’s and child’s physical changes and issues with the participating family, as well as psychological and social environmental issues. The mother-visitor relationship was a major focus during the first weeks of the intervention.

Three intervention manual themes were totally absent from the prenatal case notes: (1) Health during pregnancy; (2) Early child development; (3) Postpartum mood changes. Three themes that emerged from the case notes but that did not figure in the program manual were:

#### Discussing immigration, unstable employment and financial related issues

*« Ms X » actually lives at a friend’s house after having been kicked out of her previous home by her father’s cousin; her parents and many of her family members live in French Guiana; « Ms X » arrived in Paris about a year ago to begin vocational training.*

#### Discussing family relationships and dynamics

*Her mom arrives and joins us. She tells me that the beginning of her daughter’s pregnancy was difficult. « Ms X » agrees and tells me that she was actually at her worst at that time because she was very afraid of her mother’s reaction. So « Ms X » hid her pregnancy from her mother for 4 months.*

#### Discussing maternity services

*She was not satisfied with the consultation she had with her anaesthetist because « she [the anaesthetist] did not explain anything well, I did not understand the words she was using. » So we spent the rest of the visit reading and discussing the family worksheets on childbirth and the epidural. I noticed she was more relaxed.*

### 0–6 Months Period

In the 0–6 months period, the home visitors had to observe the mother’s adaptation to the baby, her psychological needs. They were asked to investigate the job/education-related needs, as well as couple needs. Information was to be given relatively to health education, to the baby’s early development, to the importance of social support and to the main adaptation of the home environment. Finally, home visitors were asked to counsel parents’ on their first interactions with their children.

Table 3 presents the categories from the 0–6 months intervention period. Results show that home visitors did not address two specific topics: (1) Information on health behaviors/Health education and (2) Adaptation of home environment to the baby. These missing results are coherent with the prenatal findings however three new themes were observed:

#### Home visitor observation of maternal parenting

*As I arrive, she gives her son a bath, which as a matter of fact she does very well, her gestures are self-assured, she is very conscientious.*

*When we are together with her child, « Ms X » tries to make him burp for ten minutes. She does not speak to the baby but she is very gentle with him.*

#### Counselling on administrative needs

*We arrive at the Prefecture. I distract the little one in her stroller while « Ms X » gets instructions. All of a sudden, she is speaking very quiet voice and searching for her words, she is nervous. I wait to see how she gets out of it and, discretely asking her permission to help out a little bit, I add in two or three pieces of information. I quietly reassure « Ms X, » because I sense that she is anxious and the receptionists are unpleasant, which destabilizes her.*

#### Counselling on self-esteem

*The baby’s awakening is the occasion to observe « Ms X ‘s » sensitivity to her daughter’s signs: a validation of her maternal skills and of the adjustments she has made over the past several weeks.*

*« Ms X » is interested by the rubric « baby’s health. » She explains to me that she is not comfortable taking [her daughter]’s temperature. I listen to her concerns about hurting the child and I validate her a lot.*

### 6–15 Months Period

The 6–15 months period was principally dedicated to support the development of mother-child attachment relationship. Routine thematics were to be evaluated (psychological needs, health needs, medicosocial network), discussed (couple needs and educational plans) or supported by the home visitor information (importance of partner support, adaptation of home environment, child’s developmental stages). Counselling targeted mother self-esteem and elaboration of personal goals as well as developing strategies to set limits to her child. Home visitors actively helped to find child care, if necessary.

Table 4 shows the qualitative outcomes from the baby’s 0–6 months intervention period. Two out of the sixteen themes from the CAPEDP curriculum did not appear in the case notes: (1) The mother’s educational plans for her baby and (2) Support from the mother’s partner. In contrast, both Alceste and thematic analyses identified counseling on socio-administrative problems as an important topic discussed during the intervention despite not being addressed in the CAPEDP curriculum.

### 15–24 Months Period

The last period, from the baby’s 16^th^ to 24^th^ month focused on parent’s empowerment and autonomy. Besides thematics that were routinely addressed (observing health and mental health needs and social network, discussing family’s social support network, informing on child development), home visitors were to discuss representations of parental authority, to inform on the importance to play with the child and to counsel on the way to set limits. They were also asked to promote the family inclusion in medical and social services.

Table 5 presents the outcomes from the 15–24 months period. (1) Encouragement to play with the child and (2) Organization of the mother’s own schedule to take some time off for her were the two topics from the curriculum that didn’t appear in the qualitative analyses. In this last period, five themes were broached:

#### Language development concerns

*She babbles a lot but I don’t recognize any of her words. I think I will address this subject with « Ms X » at our next visit, so that I can give her some ways to identify even a subtle retardation in her daughter’s language development, solutions that she might want to think about, and tell her some consequences of language difficulties when they are not taken into account in a young child.*

#### Feedback on the home-visiting program

*We summarize the last two years that we spent together; I say how difficult it is for me to say good-bye, I go back to how much she and her son have evolved since the time that we met, validation and telling little anecdotes. I talk about her current situation, of my confidence in her capacities and determination. I thank her as well for all that she has given to me.*

#### Social and professional situation

*She explains to me her son’s first days in child care; « Ms X » is very satisfied, happy to thus have greater availability for pursuing different approaches in professional re-integration. « Ms X » has to meet with an Advisor in order to decide how to begin her professional training, which had been interrupted by her pregnancy.*

*Health of the second child (i.e. child not directly receiving the intervention)*

*Her second son is well (…) She rarely calls him by his first name (she is reluctant to use the name chosen by his father) but speaks of him in saying « the other baby, the new baby »; I am wondering about the investment of this second child.*

#### Problems with Romantic Partner/Child’s Father

*What « Ms X » about her marital situation is still just as worrying, the situation is becoming worse. For a long time she tells me about the most recent events, her annoyances, telling me the details and her greatest fears.*

### Other Themes

Four further themes appearing in all four child age categories were identified by both thematic and computerized analyses:

#### The negative impact of the family’s social environment on the quality of the intervention

Due to the low socio-economic status and changing living conditions of many CAPEDP participants, visitors expressed difficulties organizing home visits and intervening according to the CAPEDP curriculum. The frequency of visits and the structure of each visit were disrupted by the social situation of the families.

*The last contact I had with « Ms X » over the telephone was three months beforehand; her telephone plan is suspended; during this time, I try regularly to contact her, contacting her mother who tells me that she gives my messages to her daughter, I contacted « Ms X’s » social services […]. I also send two postal letters to « Ms X, » the end of my intervention is approaching… I suspect that I won’t see the mom and child before the child turns two years old.*

*I am rapidly realizing that « Ms X’s» living situation is very insecure without any financial help except for the support of a few friends and some food and diapers for the baby which are still insufficient. […] After learning about her difficulties, I now feel uncomfortable that I had accepted a glass of orange juice.*

*I am apprehensive to invest in a two-year relationship in thinking that she can end it at any moment. « Ms X » is actually waiting (for the third time) for the ruling on her request for political asylum. I think that my attitude in this situation will be more or less to see from day to day what happens without putting long term things in place.*

#### Home visitors’ preoccupations about maternal parenting

Home visitors frequently expressed in the case notes their concern about the parenting they observed.

*She does not give her daughter a stable and coherent response. I feel frustrated in my work: we don’t have real time to talk, I don’t observe any progress and signs from the baby, « Ms X » has to handle a tired and exasperated little girl…I finish by shortening the visit, because I sense that she is overwhelmed and that she wants to calm her daughter.*

*Two times I felt uncomfortable when « Ms X » held the baby. She has her child sit on the couch and then holds her under her armpits. Nothing is holding up her head which is falling forward.*

#### Logistic constraints of the home visit

The domestic setting of home visits impacts logistical aspects of a professional relationship with families. While home visits give more control to the family, they also create problems for the home visitor who is striving to achieve distinct objectives. Most of these problems concerned home visitors having difficulty maintaining the participant’s attention or discussing the intervention objectives. They also concerned unpredictable living conditions.

*The television is turned on and fixed to an image of a DVD which plays in circles and bothers me quite a bit because it is very loud. I have to concentrate on myself in order to endure the noise.*

*I don’t know if it’s because I am pregnant and more sensible to odours and dirtiness but I felt an immediate repulsion to this future mom.*

#### Difficulties in maintaining the relationship

Home visitors frequently expressed that families were unreliable in maintaining regular visits. Home visitors reported difficulties in scheduling visits and in accessing participants, even when mothers confirmed visits. They expressed feelings of discouragement and irritation.

*I finally succeed in seeing « Ms X » after 2 planned visits that she missed and 4 cancelled visits (always with an excellent reason that does not convince at all, cancellations which annoy me).*

*I have not seen her for a month and a half. I feel the connection with her is getting more and more over-stretched, and her missed visit upset me all the more in that she did not return my phone call.*

## Discussion

### Developing a Method to Address Fidelity in Home-visiting Programs

The current study presented a method for assessing fidelity in a French home-visiting program targeting families with low socio economic status.

To evaluate the discrepancies between what the intervention intended to offer and the services the participating families effectively received, we conducted a qualitative analysis of 1,058 case notes from 105 families, which had been written by home visitors. We compared a computer-assisted textual analysis to a thematic analysis performed by researchers who were unfamiliar with the CAPEDP training manual. Then we created tables to compare the contents of (a) the program manual, (b) the thematic analysis and (c) the computer-assisted analysis.

We learned from this study that complete fidelity to the program’s curriculum could not be achieved with this study’s sample of high-risk families. Following Kitzman, Cole, Yoos, & Olds [23] and Saylor & Duggan [30], we identified inconsistencies between the intended intervention and the applied home visit intervention.

We chose to present those discrepancies in two categories (see table 6):

**Table 6:** Synthesis of the results.

Omitted thematics: not addressed while expected	Additional thematics: addressed while not expected
Prevent postpartum depression by observing mood changes	Discuss social, cultural and administrative issues
Prenatal information on early child development	Discuss the mother’s relationship with her family
Health education	Discuss relations with other services
Information on the adaptation of the home environment to the child	Discuss the problems with the partner
Discuss mother’s educational plans	Discuss the issues related to the 2^nd^ child
Inform the family on the importance of partner support	Observe the quality of maternal behaviors
Inform about the importance of playing with the child	Observe problems in the development of the child’s language
Help the mother to organize her personal schedule	Feedback on intervention

- The objectives of the intervention that were not addressed during the actual home visits despite expectations outlined in the home visitor training manual.
- The aspects of the intervention that home visitors applied despite their absence from the program’s training manual

### Objectives not Addressed During the Home Visit Intervention

The intervention manual drew upon the experience of health promotion programs which were offered to less vulnerable populations. The CAPEDP program differed from many other health promotion programs in that participants were facing very challenging social situations. Almost half of the recruited families were eligible for health services financed entirely by the French government. The focus on the mental health of mothers and their new-born children as well as on their relationship were two additional aspects that distinguished the CAPEDP program from other home visit programs. Hence, the intervention was slightly modified by the home visitors’ educational training in psychology. This affected the fidelity to the curriculum in two ways:

#### Difficulty addressing health education topics

Although the training manual urged home visitors to discuss and counsel participants on health-related behaviors during pregnancy, according to analysis of the case notes, home visitors did not address this issue. The absence of health education from case notes may be explained in three ways: First, we speculate that home visitors and families prioritized social and material issues over health education due to the urgency of social and material issues. Secondly, participants received a mean of 3 prenatal visits (SD = 2.0; 0–11, i.e. 55,3% of the intended number of visits). These 3 prenatal visits most likely sufficed in negotiating and shaping the objectives of the intervention and the relationship with the family, but were likely not substantial enough to negotiate and implement a health education intervention. Lastly, the home visitors training as psychologists may have led them to focus on mental health support rather than on health education to families.

#### Social emergencies prioritized over health-related topics

Given the many social adversities confronted by CAPEDP participants, social-related topics were the focus of most home visit conversations. While social-related topics were expected to be a secondary objective of the intervention, it became the intervention’s main focus for a significant number of the families. As a consequence, health, relational and educational issues became secondary themes in the intervention.

### Themes Addressed while not Expected

#### The psychologist-mother relationship

Although most home-visiting programs employed nurses as home visitors, the CAPEDP team, seeking to specifically address attachment and the mother-child relationship, consisted of 9 clinical and developmental psychologists. Despite the lack of health education topics in case notes, the home visitors focused particularly on building an alliance with the families, and particularly the mothers. Consequently the themes “discussing the home visitor-mother relationship” and “home visit feedback from the mother” emerged frequently across case notes from each of the four intervention periods. This finding can be assimilated to what Kitzman et al. identified in the NFP Memphis trial as a barrier to program implementation (gaining and maintaining access to the family) [23].

#### The home visitor as evaluator

The home visitors provided frequent feedback concerning:

- Preoccupations about the quality of maternal parenting
- Problems in child language development

Our thematic analysis revealed that these two categories appeared within the “observe” category. This means that the home visitor provided feedback to the case note reader but did not directly address these subjects with the family.

#### Social issues

The lack of material and social resources in participating CAPEDP families was a major focus of the intervention. Home visitors thus often discussed a family’s social and administrative problems first, before focusing on the health promotion/prevention contents of the intervention. These findings are consistent with results from Darius Tandon et al. [30] and Hiatt, Sampson & Baird [29] researches, while this latter study used paraprofessionals to run the preventive intervention.

Immigration to France and assimilation to French culture were also discussed, often alongside the issue of social isolation. Lastly, discussing the family’s relationship with other services, such as the Maternal and Infant Protection Agency services (*Protection Maternelle et Infantile*) was on the home visitors’ agenda, as the CAPEDP preventive intervention could not meet all of the family’s needs.

#### Relationship with the family

The mother’s relationship with her partner or the child’s father were recurrent themes in the intervention (particularly when the relationship was conflictual). Romantic relationships and difficulties in the relationship with the family were also discussed. Finally, the relationship between the mother and additional children she may have had within the time of the intervention, constituted an additional topic for home visitors.

### Perspectives

The CAPEDP project intended to bring out the expertise of psychologists within a home visitation intervention. This decision to hire psychologists to assume the role of home visitors impacts how mental health professionals can conceptualize traditional psychological interventions as well as the paradigm for home visit interventions.

With regards to conventional psychological interventions in France, CAPEDP, by using a home visitation protocol, allowed professionals to develop their relational skills within an ecological context. It enabled new psychological practices in the field of prevention to be sketched out.

On the other hand, the results from this qualitative study question the idea of having a homogeneous team of home visitors in terms of their backgrounds. As Darius Tandon and colleagues stressed, home visitors trained to be health care providers can be unsettled by the social situation of the families [30]. In this study, we identified that the major themes of home visits fell within a triangular model of social, psychological and health issues to address. While our research team focused on psychological issues, future home visitation interventions should provide multidisciplinary training to the professionals they select to be home visitors. This training should address a diversity of issues relevant to the targeted population and will ultimately enhance the global efficacy of the home visit intervention. Investigators should also consider offering a multidisciplinary supervision to the home visitors, both by seniors practitioners from the same field (nurses for NFP-like interventions [13], psychologists in CAPEDP) and by other professionals, such as social workers.

Results from the CAPEDP trial and from this qualitative study will help mental health professionals understand mechanisms underlying a home visit intervention that was led by psychologists and to judge the extent to which psychologists can impact the social and health conditions of vulnerable families through home visits.

### Conclusion

We presented a qualitative evaluation of a home-visiting program’s adherence to its original protocol in Paris, France from 2006 to 2011. The use of two qualitative methods (thematic/textual) was developed to analyze 1,058 home visit case notes from 105 families, written by the home visitors. We learned from this study that the home visitors partially followed the intervention’s original curriculum. Several of the program’s objectives were not addressed, mainly because of the urgent needs of the participating families that took precedence over certain program objectives. While confronted with practical issues, it is necessary to think about the malleability of the structure of such a home-visiting *program*. In the present program, home visitors expressed difficulties addressing unexpected topics and the absence of several themes that they could not address (prevention of postnatal depression, child development, etc.).

Home visitation programs should allow for enough flexibility in the intervention for home visitors to adapt their visits to the needs of families. Adapting the intervention can be especially helpful when families live in impoverished or otherwise aversive social conditions and have urgent needs related to these circumstances. Future home visitation programs should use a strategy in which home visitors partially negotiate the intervention with the family. They should also adapt their curriculum to the reality and the social, psychological and health needs of the targeted population to ensure that the intervention has an impact on the individual and the community. In light of these findings, the professional background of home visitors should be considered carefully and alongside the targeted population’s needs. The training of professionals’ should be ongoing, provided prior to beginning the intervention, and at designated time points throughout the intervention. Home visitors in this study stated that it was difficult to address social issues presented by participating families. This feedback highlights the importance of continuous training, in which home visitors would be trained to address challenges as they arise from the intervention. In the current study, psychologists were hired to serve as home visitors. However the benefits of interventions using mixed teams formed with social workers, psychologists and nurses, remains unknown and should be the object of future research.
